# Sensitivity of ISLT to memory impairment in prodromal Alzheimer's disease is improved by consideration of age and sex

**DOI:** 10.1002/alz70857_103927

**Published:** 2025-12-25

**Authors:** Paul Maruff

**Affiliations:** ^1^ Cogstate Ltd., Melbourne, VIC, Australia; Turner Institute for Brain and Mental Health, School of Psychological Sciences, Monash University, Melbourne, VIC, Australia; The Florey Institute of Neuroscience and Mental Health, The University of Melbourne, Parkville, VIC, Australia

## Abstract

**Background:**

The International Shopping List Test (ISLT) is sensitive to episodic memory dysfunction in symptomatic Alzheimer's disease (AD). it is applied in clinical contexts where performance of individuals with suspected AD is compared to normative data. Current ISLT normative data is stratified by age only (declines). The study measured the sensitivity of the ISLT performance measures in a well‐characterized prodromal AD cohort using existing normative data and updated normative data that stratified data according to age (5‐year‐strata) and sex.

**Method:**

321 Adults with MCI (age 65 to 80yrs, CDR=0.5) were drawn from the screening visit of those with abnormally high amyloid (AB+) determined from PET scan or CSF sampling for entry into the MissionAD elenbecestat program. Normative data consisted of 500 older cognitively unimpaired (CU) adults from AIBL study for whom PET or CSF sampling showed normal AB levels. Associations between ISLT scores and years of education were investigated using correlation. For each MCI participant, ISLT immediate and delay, and immediate plus delay scores were standardized using the group means and standard deviations from the current ISLT, and updated ISLT norms. Sensitivity was defined as the proportion of the AB+MCI and AB‐ MCI groups classified with a standardized score </=‐1SD (e.g. 1‐specificity = 87%). Sensitivity was computed and compared using McNemars test for the existing age (decile), and new age (quintile) x sex adjusted normative data.

**Results:**

There was no influence of education level on ISLT immediate or delated scores in the CU (*r* = 0.16, *p* = 0.34) or total MCI (*r* = 0.09, *p* = 0.34) group. Sensitivity to MCI related memory dysfunction was improved significantly from that associated with current norms through comparison with sex and 5‐year age stratified norms (recall 83% to 91% (*p* <0.05), delayed recall 81% OR 91% (*p* <0.01), recall=delated recall 87% to 97% (*p* <0.01).

**Conclusion:**

The sensitivity of the ISLT to memory dysfunction in MCI due to AD is improved by addition of information about sex and 5‐year age strata to normative data.

